# Variable anisotropic FOV for 3D radial imaging with spiral phyllotaxis (VASP)

**DOI:** 10.1002/mrm.28449

**Published:** 2020-08-27

**Authors:** Guruprasad Krishnamoorthy, Jouke Smink, Joao Tourais, Marcel Breeuwer, Marc Kouwenhoven

**Affiliations:** ^1^ Department of MR R&D–Clinical Science Philips, Best The Netherlands; ^2^ Department of Biomedical Engineering Eindhoven University of Technology Eindhoven The Netherlands

## Abstract

**Purpose:**

To develop a new 3D radial trajectory based on the natural spiral phyllotaxis (SP), with variable anisotropic FOV.

**Theory & Methods:**

A 3D radial trajectory based on the SP with favorable interleaving properties for cardiac imaging has been proposed by Piccini et al (*Magn Reson Med*. 2011;66:1049‐1056), which supports a FOV with a fixed anisotropy. However, a fixed anisotropy can be inefficient when sampling objects with different anisotropic dimensions. We extend Larson’s 3D radial method to provide variable anisotropic FOV for spiral phyllotaxis (VASP). Simulations were performed to measure distance between successive projections, analyze point spread functions, and compare aliasing artifacts for both VASP and conventional SP. VASP was fully implemented on a whole‐body clinical MR scanner. Phantom and in vivo cardiac images were acquired at 1.5 tesla.

**Results:**

Simulations, phantom, and in vivo experiments confirmed that VASP can achieve variable anisotropic FOV while maintaining the favorable interleaving properties of SP. For an anisotropic FOV with 100:100:35 ratio, VASP required ~65% fewer radial projections than the conventional SP to satisfy Nyquist criteria. Alternatively, when the same number of radial projections were used as in conventional SP, VASP produced fewer aliasing artifacts for anisotropic objects within the excited imaging volumes.

**Conclusion:**

We have developed a new method (VASP), which enables variable anisotropic FOV for 3D radial trajectory with SP. For anisotropic objects within the excited imaging volumes, VASP can reduce scan times and/or reduce aliasing artifacts.

## 1 INTRODUCTION

Recent advancements in MR hardware and software have led to increased interest in the clinical use of cardiac MRI (CMR) for heart disease diagnostics. This noninvasive technique provides excellent soft tissue contrast and enables the assessment of cardiac morphology and function. However, 2 shortcomings of the currently used methods in routine clinical practice are: (1) the complex, extensive, and time‐consuming planning before the actual image acquisition; and (2) the required patient cooperation to perform breath‐hold imaging.^1^, ^2^ For these reasons, performing comprehensive CMR is limited to experienced operators. Free‐breathing, volumetric CMR with high, isotropic resolution was proposed to overcome the above‐mentioned shortcomings.^3^ This technique simplifies the scan planning significantly by eliminating the need for angulation, and it also eliminates the need for breath‐hold by performing a respiratory gated acquisition at the cost of increased total scan time. Acquisition and reconstruction techniques that improve the respiratory gating efficiency while maintaining the above said desired features have received considerable attention in recent years.^4^, ^5^, ^6^, ^7^ In particular, 3D projection reconstruction (3DPR) has been proposed as a robust acquisition technique for CMR.^8^ 3DPR is intrinsically robust to motion and flow artifacts. The point spread function (PSF) of 3DPR is robust to undersampling in all 3 directions because the aliasing artifacts in 3DPR appear more like benign pseudo‐random noise or streaks when compared to classical fold‐over aliasing in Cartesian sampling. Thus, scan‐time reduction for 3DPR can be achieved using a FOV that is significantly smaller than the object inside the excited imaging volume while maintaining acceptable image quality.^9^ Information about the respiratory and contractile heart motion can be extracted from the radial projections itself for self‐navigated reconstruction.^10^, ^11^

Because of the required cardiac synchronization, a CMR acquisition is typically performed in a highly segmented/interleaved fashion. When interleaved, 3DPR is used in combination with a balanced steady‐state free precession (bSSFP) technique, the k‐space trajectory should be designed such that the angular distance of the radial projections between the successive excitations (in each segment) are minimized. Otherwise, the rapidly changing gradient moments caused by the trajectory can induce increased eddy currents in the conductive components of the MR system. These induced eddy currents can disturb the perfectly balanced acquisition scheme and cause image artifacts.^12^ To minimize these artifacts, Piccini et al proposed a 3DPR acquisition based on the natural spiral phyllotaxis (SP), with interleaving properties beneficial for the bSSFP technique and cardiac imaging.^13^ When the total number of interleaves is a Fibonacci number, the SP arranges the trajectory in such a way that (1) the angular distance between successive projections in each interleave is minimal; and (2) each new interleave is placed according to the golden ratio in the azimuthal direction.

One of the limitations of the conventional SP is that it only supports a FOV with a fixed anisotropy, which can lead to a relatively low scan efficiency if the aspect ratio of the object in the excited imaging volume does not match the FOV anisotropy. Larson et al proposed a method of designing trajectories with varying angular densities corresponding to the anisotropic FOV, resulting in scan time reductions without increased aliasing artifacts for both 2D and 3DPR radial imaging.^14^ This method did not yet take into account golden‐angle view ordering,^15^ which has since become a widely used acquisition scheme in dynamic imaging. Wu et al later proposed a method to support anisotropic FOV for golden‐angle 2D radial imaging, which preserved the desired nonuniform angular densities for arbitrary temporal window length and demonstrated the improved scan‐efficiency.^16^ Whereas this method can easily be extended to 3D stack of stars, it cannot be applied to conventional SP (3DPR) because: (1) SP is defined by 2 angles (azimuthal and polar), applying it only to the azimuthal angle leads to aliasing within the desired FOV^14^; and (2) applying it only to the polar angle results in a significant deviation from the desired anisotropic FOV because the polar sampling distribution is not uniform in conventional SP.^16^

In this study, we applied Larson’s 3DPR method to enable variable anisotropic FOV for spiral phyllotaxis (VASP). The proposed VASP technique maximizes scan efficiency, while retaining the favorable interleaving properties of conventional SP. The benefits of VASP are demonstrated through simulations, phantom experiments, and in vivo cardiac imaging.

## 2 THEORY

In 3DPR, a 3D spherical coordinate system can be used to define the redouts by the azimuthal angle (*ϕ*), polar angle (*θ*), and the radius of each radial projection (*k*_max_), where *ϕ* is the deflection in the *k_x_*–*k_y_* plane relative to the positive *k_x_* axis, and *θ* is the deflection from the positive *k_z_* axis in k‐space. Uniform sampling of k‐space on a sphere with constant angular density and constant *k_max_* generates a near‐spherical FOV with isotropic resolution. If an anisotropic FOV is desired for 3DPR, the FOV in *x* − *y* plane (*FOV_ϕ_*), the FOV in (oblique) *z* direction (*FOV_θ_*), and the spatial resolution (*res*) can be expressed as a function of *ϕ* and *θ*, as shown in the following equations^14^:

(1) $$FOV_{\phi }\left(\theta =\frac{\pi }{2},\phi +\frac{\pi }{2}\right)=\frac{1}{\Delta k_{\phi }\left(\phi \right)}$$

(2) $$FOV_{\theta }\left(\theta +\frac{\pi }{2},\phi \right)=\frac{1}{\Delta k_{\theta }\left(\theta \right)}$$

(3) $$res\left(\theta ,\phi \right)=\frac{1}{2k_{\max}\left(\theta ,\phi \right)},$$

where $\Delta k_{\phi }\left(\phi \right)$ and $\Delta k_{\theta }\left(\theta \right)$ are the azimuthal and polar angular spacing at a given *ϕ* and *θ,* respectively. In the conventional SP,^13^ $FOV_{\phi }$ and $FOV_{\theta }$ are governed by the total number of radial projections N. The azimuthal and polar angles, which are generated for each projection *n* as

(4) $$\phi \left(n\right)=\frac{2\pi }{360}\;\cdot \;n\;\cdot \;\varphi_{\mathrm{gold}}$$

(5) $$\theta \left(n\right)\;=\;\frac{\pi }{2}\;\cdot \;\sqrt{\frac{n}{N},}$$

where $\varphi_{\mathrm{gold}}\approx 137.51^{\circ },$ *n* = 0, 1, 2, … *N‐1*. As Piccini et al already showed, if the acquisition is performed in an interleaved fashion, Equations (4) and (5) can be formulated for each projection *r* within interleave *i* as

(6) $$\phi \left(i,r\right)=\frac{2\pi }{360}\;\cdot \;\left(i+\left(r*I\right)\right)\;\cdot \;\varphi_{\mathrm{gold}}$$

(7) $$\theta \left(i,r\right)=\frac{\pi }{2}\;\cdot \;\sqrt{\frac{\left(i+\left(r*I\right)\right)}{N}},$$

where *I* = number of interleaves, *R* = number of projections per interleave, R∗I=N, i=0,1,…I‐1, and r=0,1,…R‐1. When the number of interleaves I is a Fibonacci number, the trajectory arranges itself in such a way that, (1) within each interleave, the tip of the first projection is close to the north pole, and the tip of successive projections traverse smoothly to the hemisphere with minimal angular distance; and (2) each new interleave is placed in the largest azimuthal gap left by the preceding interleave according to the golden ratio. These properties are particularly useful for bSSFP technique with cardiac imaging.

In the proposed VASP method, to maintain the interleaving properties of the SP, the azimuthal angle was generated using Equation (6), whereas the polar angles and the total number of projections N are adapted to create an anisotropic FOV, with variable anisotropy in the z direction. To compute these polar angles, the proposed method takes the input of FOV in x‐y plane with $FOV_{x}=FOV_{y}$ ($FOV_{\mathrm{xy}}$), FOV in *z* direction ($FOV_{z}$), FOV shape ($FOV_{\theta }\left(\theta \right)$) in polar direction, and the required isotropic resolution $\left(res_{\mathrm{xyz}}\right)$. An initial set of polar angles *θ* is iteratively computed for index m using Larson’s method based on the following equation:

(8) $$\theta \text{[m + 1}]=\frac{1}{k_{\max}*FOV_{\theta }\left(\theta \text{[m] +}\;\frac{\pi }{2},FOV_{z},FOV_{\mathrm{xy}}\right)}+\theta \text{[m] ,}$$

where θ[0]=0, *m* = 0, 2, … *M‐1*, $k_{\max}=\frac{1}{2res_{\mathrm{xyz}}}$, and $FOV_{\theta }$ can be any convex shape function. M is the total number of polar angles and is defined by the number of iterations (m) required for θ[m] to reach $\frac{\pi }{2}$. To improve the quality of the trajectories, fine adjustments are done on θ[m], as described in detail in Ref. 14. To create a continuous sampling path in the polar direction, the initial set of coarsely computed polar angles *θ* is linearly interpolated to obtain the final set of densely sampled polar angles, θ. This linear interpolation uses $N_{\mathrm{est}}\left[m\right]$ polar angles (θ) between θ[m] and θ[m + 1]. $N_{\mathrm{est}}\left[m\right]$ is computed using the following formula:

(9) $$N_{\mathrm{est}}\left[m\right]=\frac{FOV_{\mathrm{xy}}}{res_{\mathrm{xyz}}}*\pi \text{*sin}\left(\frac{\theta \text{[m] +}\;\theta \text{[m}+1])}{2}\right),$$

where $N_{\mathrm{est}}\left[0\right]=1$.

The total number of radial projections (N) to be acquired is computed as

(10) $$N=\sum_{m=1}^{M}N_{\mathrm{est}}\left[m\right].$$

For each readout r within interleave *i*, the polar angular index n is computed as *n* = $\left(i+\left(r*I\right)\right)$.

## 3 METHODS

### 3.1 Numerical simulations

Based on numerical simulations, 5 analyses were made. The aim of the first analysis was to estimate the potential origin of eddy currents due to the trajectory within the interleave for VASP and conventional SP. Thus, the average angular distance between the successive radial projections within 1 interleave was computed as a measure of changing gradient moments. This was done for a different Fibonacci number of interleaves and projections as well as for several different FOV anisotropies.

The aim of the second analysis was to compare the spatial distribution of projections in k‐space for conventional SP and VASP with several different FOV anisotropies. To do this, Voronoi’s approach was used to measure the area occupied by each projection on the surface of a sphere. This area was measured with a total number of 12,818 projections for all the 3DPR methods, and the measured area was normalized so that the distributions can be compared.

The third analysis aimed to compare the PSF of conventional SP and VASP with different FOV anisotropies. All the PSFs were generated with identical resolution and displayed on a logarithmic scale.

The fourth analysis aimed to demonstrate the level of aliasing artifact of VASP on a 3D Shepp‐Logan numerical phantom^17^ of matrix size 192^3^. The 3D Shepp‐Logan phantom was cropped in the *z*‐direction to simulate ideal volume selective excitation in the transverse plane. The images were reconstructed on a 384^3^ Cartesian grid.

The aim of the fifth analysis was to quantify the scan‐time benefit of variable anisotropic FOV. The total number of radial projections required for conventional SP was compared against VASP for different FOV anisotropies.

All the analyses were performed in MatLab R2018b (Mathworks Inc., Natick, MA) using in‐house–developed code.

### 3.2 MR experiments

The VASP method was implemented on a 1.5T Ingenia Scanner (Philips, Best, The Netherlands). Phantom images were acquired with a bSSFP sequence using both conventional SP and VASP with different FOV anisotropies. Imaging parameters were: TR/TE: 3.9/1.7 ms, flip angle: 90⁰, bandwidth: 868 Hz/px, isotropic acquisition resolution: (1.25 mm)^3^, and a radial FOV of 240 mm. A short volume selective RF pulse with a heavily truncated sinc envelope with a duration of 0.85 msec was used to excite an axial slab with a width of 150 mm. In vivo cardiac images were acquired in axial orientation using a bSSFP sequence in a healthy volunteer under a protocol approved by the ethics committee with written informed consent. Imaging parameters were the same as for the phantom experiment, except for the following parameters: The acquisitions were electrocardiogram‐triggered using a mid‐diastolic trigger delay. A total of 233 interleaves with 34 projections per interleave was used, resulting in an acquisition window duration of 132.6 ms per interleave (each preceded by 6 dummy startup cycles). The dimensions of the anatomy inside the excited imaging volume were ~430 × 280 × 150 mm^3^ (*x* × *y* × *z*) [anisotropic FOV ratio 100:48:35]. The aliasing‐free FOV of VASP was 131 × 131 × 46 mm^3^ [100:100:35]. For conventional SP, the aliasing‐free FOV was 79 × 79 × 116 mm^3^ [100:100:147]. To minimize motion due to respiration, a diaphragmatic pencil‐beam navigator was used with an acceptance gate of 6 mm, and volume tracking was performed. For a cardiac frequency of 70 beats per minute, the nominal scan time for VASP and conventional SP was 3:20 min each (excluding respiratory gating inefficiency). For both phantom and in vivo experiments, 16‐element anterior and 12‐element posterior phased array coils were used for signal reception. The signals from individual coil elements were combined^18^; and to correct for the signal uniformity, constant level appearance algorithm was applied.^19^ Density compensation in gridding was based on the algorithm proposed by Zwart et al.^20^ In‐line reconstruction was performed on the scanner using algorithms developed in Recon 2.0 (Philips, Best, The Netherlands) with a reconstruction time of ~20 s per dataset.

## 4 RESULTS

**FIGURE 1:**
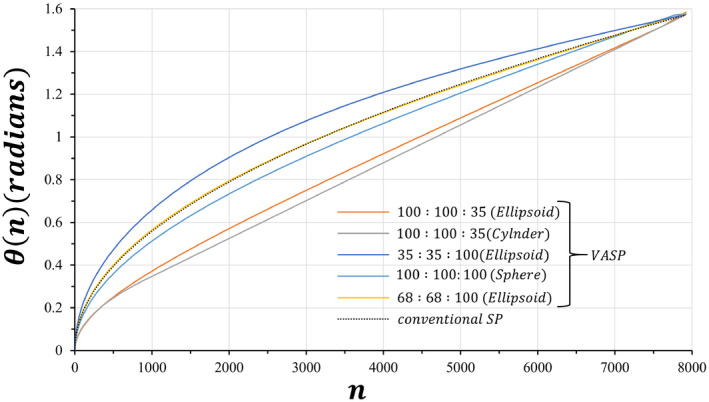
Plots of polar angles θ as a function of the projection number n, generated for conventional SP and VASP with FOV anisotropy ratios. All VASP FOV anisotropy ratios as well as conventional SP used the same number (7922) of radial projections. Due to the square root function used in conventional SP to generate the polar angles, by default SP had an anisotropic FOV that closely matched the polar angles of VASP with FOV ratio of 68:68:100 (*x*:*y*:*z*). SP, spiral phyllotaxis; VASP, variable anisotropic FOV for spiral phyllotaxis

The polar angles $\theta \left(n\right)$ with n=0,1,2…N‐1 generated for conventional SP and VASP with different FOV anisotropies are shown in Figure 1. Due to the square root function used in conventional SP to generate the polar angles, by default SP had an anisotropic FOV that closely matched the polar angles of VASP with FOV ratio of 68:68:100 (*x*:*y*:*z*).

**FIGURE 2:**
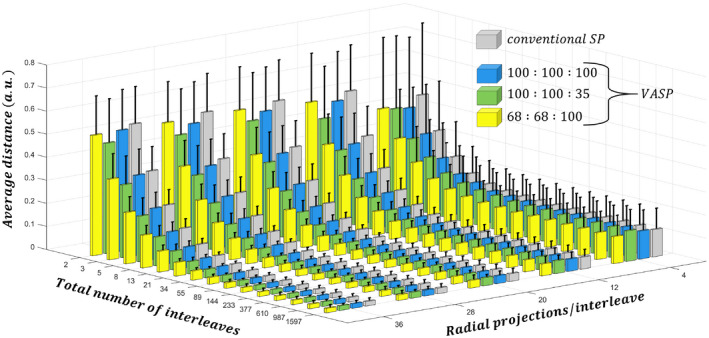
Bar plots of the average distance between the tips of successive radial projections on a sphere for conventional SP and VASP with different anisotropic FOV ratios. The SD is shown as error bars. Both methods show a similar trend of decreased tip distance as the total number of interleaves and the number of projections per interleaves increases

The average distance between the tips of successive radial projections is a measure of changing gradients moments and thus of expected eddy currents. The average angular distance for SP and VASP with different FOV ratios is shown in Figure 2. The average distance of VASP for all FOV ratios were practically similar to that of the conventional SP.

**FIGURE 3:**
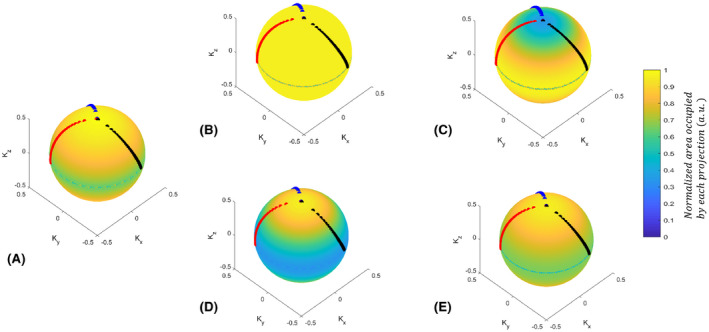
The spatial distribution of projections on a sphere for different anisotropic FOV ratios. (A) Conventional SP, (B) VASP with a spherical FOV of 100:100:100, (C) oblate spheroid with ratio of 100:100:35, (D) prolate spheroid with ratio of 35:35:100, and (E) prolate spheroid with ratio of 68:68:100. The readout tips of the first 3 consecutive interleaves (with 34 readouts per interleave, out of 377 interleaves) are highlighted in black, red, and blue, respectively. Note: VASP with (B) isotropic FOV had a significantly more uniform distribution when compared to (A) conventional SP. The distribution of projections of (A) the conventional SP had an effective FOV of 68:68:100, and it closely matched to the readout distribution of (E)

The spatial distribution of the radial projections in k‐space is shown for conventional SP and VASP in Figure 3. By default, conventional SP (Figure 3A) had an anisotropic distribution of projections that is denser in K_z_ than in K_x_ and K_y._ In contrast, VASP with a FOV ratio of 100:100:100 showed a significantly more uniform distribution of projections in k‐space (Figure 3B)_._ VASP with FOV ratios of 100:100:35 and 35:35:100 are shown in Figure 3C,D, respectively. The distribution of conventional SP closely matched the distribution of VASP with a FOV ratio of 68:68:100 (Figure 3E)_._ As can be seen in Figure 3, the VASP projection tips followed a similar trajectory as that of the conventional SP.

**FIGURE 4:**
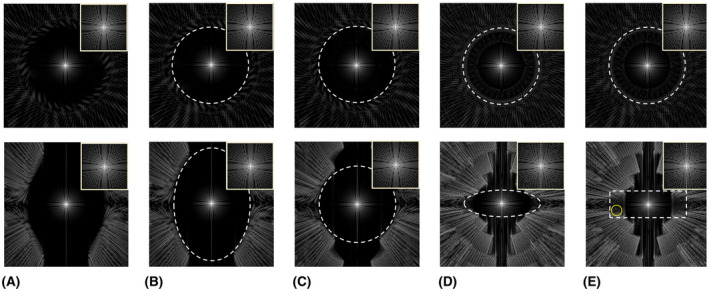
PSFs of conventional SP and VASP for different FOV anisotropies and shapes. The central slices of the transverse plane and the coronal plane are shown in the top row and the bottom row, respectively, with zoomed images of the main lobe in the top right corner of each image. The white dashed lines indicate the applied unaliased FOV, which is specified in voxel units below. PSF of (A) conventional SP with 62919 projections, (B) VASP with 62919 projections for ellipsoidal FOV of 177 × 177 × 260, (C) VASP with 49339 projection for spherical FOV of 177 × 177 × 177, (D) VASP with 22468 projections for ellipsoidal FOV of 177 × 177 × 62, and (E) VASP with 24174 projections for cylindrical FOV of 117 × 117 × 62. Note: PSF of (A) conventional SP is similar to that of (B) VASP with FOV ratio of 68:68:100. (E) PSF of VASP with cylindrical FOV produced some low‐level aliasing within the applied FOV, indicated by the yellow circle. PSF, point spread function

The PSF of conventional SP shown in Figure 4A was very similar to the PSF of VASP with a FOV ratio of 68:68:100 (Figure 4B). In Figure 4C‐E, the PSF of VASP with FOV ratio of 100:100:100 (sphere), 100:100:35 (oblate spheroid), and 100:100:35 (cylinder) are shown, respectively. For the cylindrical FOV, low‐level aliasing of intensity 9.4 × 10^−4^ was observed within the prescribed FOV (yellow circle indicated in Figure 4E) when the intensity of the central peak is normalized to 1.

**FIGURE 5:**
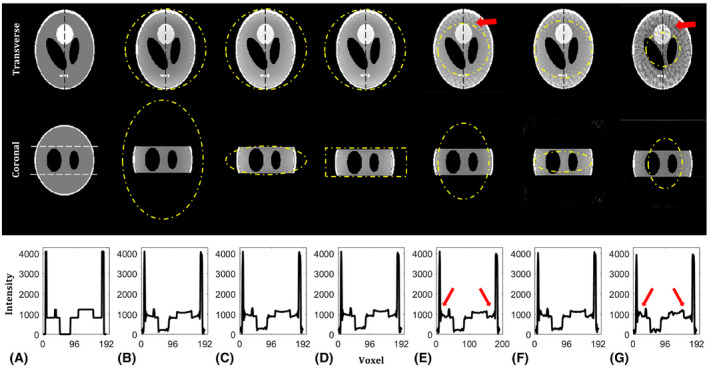
Numerical phantom reconstructions of conventional SP and VASP for different FOV anisotropies and shapes. The central slice of the transverse and coronal plane is shown in the top and middle row, respectively, with the normalized intensity profile (along the vertical dotted black line) shown in the bottom row. The unaliased FOVs are indicated by yellow dotted lines. (A) Ground truth images of the 3D Shepp‐Logan phantom used for simulation with white dotted lines in the coronal plane indicating the volume selective excitation. Images reconstructed using (B) fully sampled conventional SP with 62919 radial projections for FOV of 177 × 177 × 260, (E) undersampled conventional SP with 22468 radial projections for FOV of 106 × 106 × 156, and (G) 11234 radial projections for FOV of 75 × 75 × 110. Images reconstructed using (C) fully sampled VASP with 22468 radial projections for ellipsoidal FOV of 177 × 177 × 62, (D) fully sampled VASP with 24174 radial projections for cylindrical FOV of 177 × 177 × 62, and (F) undersampled VASP with 11234 radial projections for FOV 125 × 125 × 44. Note the increased aliasing artifacts highlighted by the red arrow in the images reconstructed using the conventional SP as compared to the VASP for a given number of radial projections

Figure 5 shows the images of a numerical phantom demonstrating the level of aliasing artifacts of VASP and conventional SP. To obtain aliasing‐free images, VASP with a FOV ratio of 100:100:35 required only 35.7% of radial projections for an ellipsoidal FOV shape (Figure 5C), and 38.4% radial projections for a cylindrical FOV shape (Figure 5D) when compared to conventional SP (Figure 5B). Aliasing artifacts were clearly visible in undersampled conventional SP (Figure 5E) with 35.7% radial projections. Images obtained with VASP (Figure 5F) show significantly reduced aliasing artifacts when compared to conventional SP (Figure 5G) for a given number of radial projections.

**FIGURE 6:**
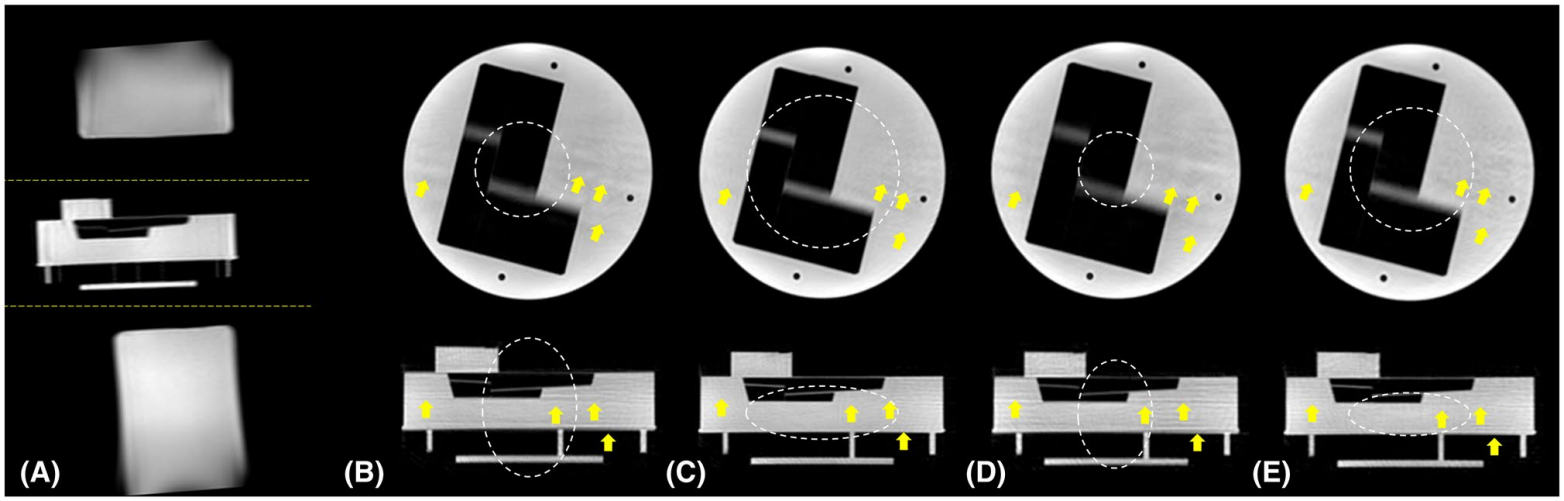
Phantom images demonstrating the performance of VASP compared to conventional SP. (A) A cylindrical phantom was placed in between 2 elongated cylindrical phantoms as shown in the sagittal scout image. The central slice of the transverse plane and the coronal plane are shown in the top and bottom row, respectively, in (B‐E). All the images in (B‐E) were acquired using a volume selective RF excitation shown by the yellow dotted lines in (A), and the unaliased FOVs are shown by white dotted lines in (B‐E). Images acquired using (B) conventional SP with 7933 projections for unaliased FOV of 78 × 78 × 115 mm^3^, and (D) conventional SP with 4896 projections for 61 × 61 × 90 mm^3^. Images acquired using (C) VASP with 7933 projections for ellipsoidal unaliased FOV of 130 × 130 × 46 mm^3^, and (E) VASP with 4896 projections for ellipsoidal unaliased FOV of 103 × 103 × 36 mm^3^. Note the reduced aliasing artifacts (highlighted by yellow arrows) in VASP when compared to conventional SP for each of the number of radial projections

Figure 6 shows phantom images for VASP and conventional SP with different aliasing levels depending on the size and anisotropy of the (unaliased) FOV. The aliasing artifacts due to undersampling were significantly reduced with VASP when the FOV anisotropy closely matched the anisotropy of the object being imaged (Figure 6C) compared to conventional SP (Figure 6B) using the same number of radial projections. For stronger undersampling, the difference between VASP (Figure 6E) and conventional SP (Figure 6D) is even larger.

**FIGURE 7:**
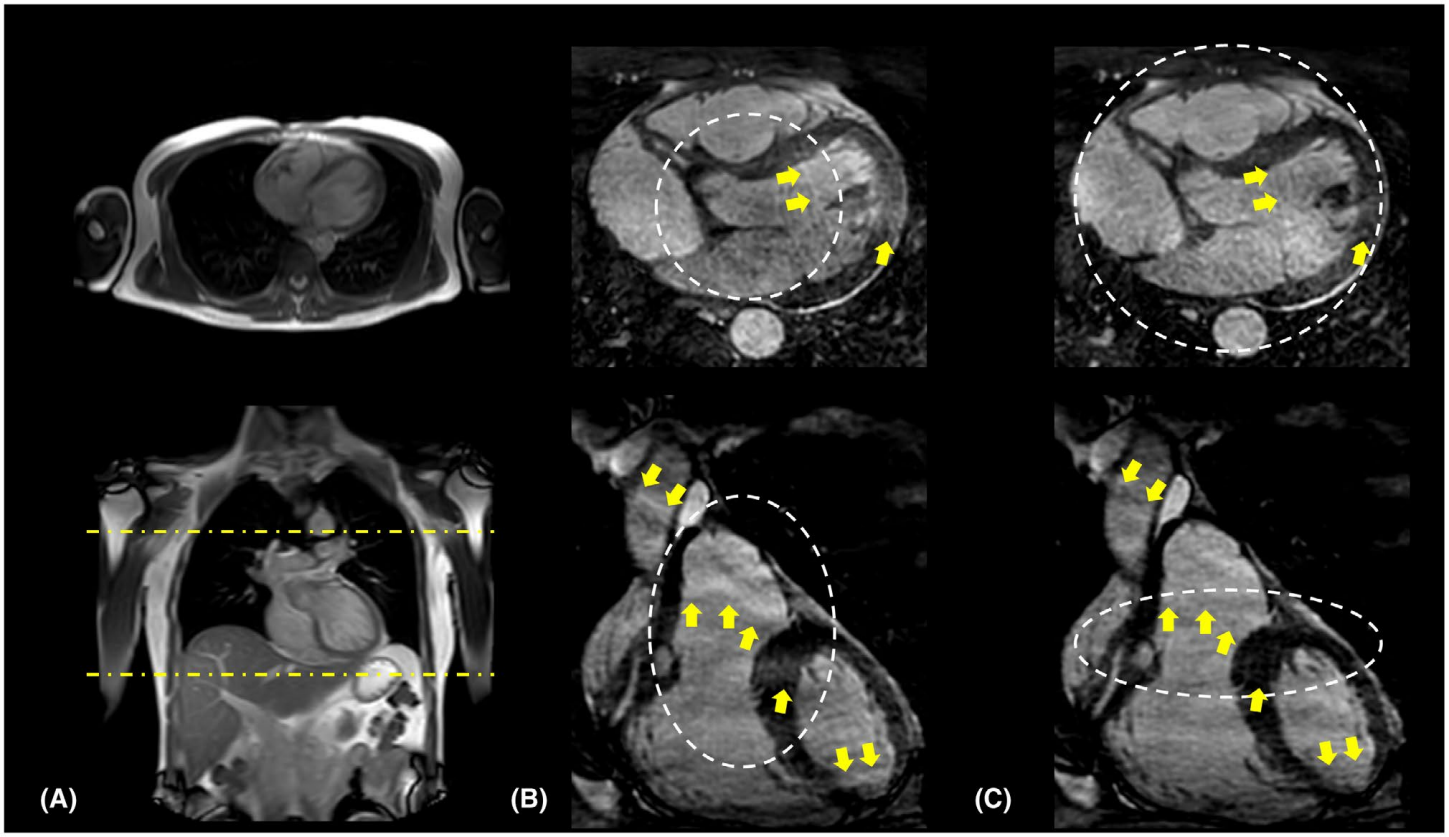
In vivo images acquired using (B) conventional SP and (C) VASP. A representative reformatted slice in the transverse plane and coronal plane are shown in the top and bottom row, respectively. 2D scout images used for planning the cardiac triggered acquisitions are shown in (A) with yellow dotted lines indicating the volume selective excitation. The aliasing‐free FOV for both methods are highlighted by white dotted lines. Note the reduced aliasing artifacts (indicated by yellow arrows) for (C) VASP when compared to (B) conventional SP as a result of adapting the FOV to match the shape of the excited volume being imaged. Images in (B) and (C) are cropped to show only the region of interest

Figure 7 compares the in vivo cardiac images obtained using the VASP and conventional SP. In line with results obtained in the numerical simulations and the phantom experiments, fewer aliasing artifacts were observed in the images acquired using VASP for the same number of radial projections when compared to the conventional SP method, as shown in Figure 7B,C.

The percentage of radial projections required in VASP when compared to the conventional SP as a function of the FOV anisotropy ratio is shown in the Supporting Information Figure S1, which is available online. For a FOV anisotropy ratio of 1.47 (68:68:100), VASP required approximately the same number of projections as conventional SP to satisfy Nyquist criteria, but for all other FOV anisotropies VASP required fewer projections.

## 5 DISCUSSION

In the current study, we report a new 3DPR design based on the VASP that retains all the interleaving properties of phyllotaxis pattern: (1) the distance between the tips of projections within each interleave is minimal (shown in Figures 2 and 3), which can be beneficial for bSSFP; and (2) successive interleaves are separated by the golden angle and can be beneficial for dynamic imaging applications.^15^

Conventional SP uses a square root function to compute the polar angles, which results in an anisotropic FOV with an approximate ratio of 68:68:100, as measured using Voronoi’s approach (Figure 3), and as also verified using PSF simulations (Figure 4). When simple density compensation was used for the conventional SP as described in Ref. 13, the reconstruction accuracy on a numerical phantom (not shown here) was suboptimal when compared to the reconstruction using iterative density compensation method proposed by Zwart et al.^20^ This is due to the anisotropic distribution of readouts in the conventional SP that was not accounted for in the simple density compensation. Hence, we chose to use the iterative density compensation throughout the study.

Whereas $FOV_{\theta }\left(\theta \right)$ (Equation 8) can be any convex FOV shape, we prefer elliptical $FOV_{\theta }$ (ellipsoidal FOV shape) because it required fewer projections when compared to rectangular $FOV_{\theta }$ (cylindrical FOV shape) with similar image quality (shown in Figure 5C,D); thus, we used ellipsoidal FOV for VASP in all the MR experiments. For most of the FOV anisotropies, VASP requires fewer projections when compared to the conventional SP (Supporting Information Figure S1). For example, images simulated using VASP required ~65% fewer projections for a FOV asymmetry of 100:100:35 when compared to conventional SP while producing virtually the same image quality, as shown in Figure 5.

In phantom experiments (Figure 6), VASP significantly reduced aliasing artifacts when compared to conventional SP for the same number of radial projections. VASP had similar aliasing artifacts with fewer radial projections when compared to conventional SP. The in vivo results (Figure 7) confirmed that VASP reduced aliasing artifacts when compared to the conventional SP for a given number of radial projections.

Figure 3 shows that the distribution of radial projections on a sphere is denser in the horizontal plane with *k_z_* = 0 for both conventional SP and VASP. As a result, there is an asymmetric distribution of aliasing energies (less along the *z*‐axis) in the PSFs, as shown in Figure 4. For a more uniform distribution of projections, alternative mathematical models could be used.^21^ However, these models are computationally intensive, making it not feasible for on‐the‐fly computation of angles based on the prescribed FOV, and are not expected to improve image quality significantly.

The combination of parallel imaging, constrained reconstructions, and golden‐angle radial sampling has gained considerable attention recently to push the limit of accelerating CMR.^22^, ^23^ VASP can be easily incorporated into these frameworks to substitute the conventional SP to further reduce imaging times, depending on the anisotropy of the object dimensions within the excited imaging volume. The proposed method only has small computational demands and can be computed on the fly for exam specific anisotropic FOVs.

The proposed VASP method was developed for full‐projection (full‐echo) design; however, it can be easily modified for half‐projection design such as UTE,^24^ zero TE,^25^ water‐ and fat‐suppressed proton projection MRI,^26^ and pointwise encoding time reduction with radial acquisition.^27^ This can be achieved by designing the set of polar angles (θ[m]) between 0 to *π* instead of 0 to $\frac{\pi }{2}$, based on the prescribed FOV using Equation (8). To create a full sampling path in polar direction, the initial set of polar angles can be linearly interpolated to θ[M + 1]=π, for which M is the total number of initial sets of polar angles designed using Larson’s method. The azimuthal angle can be computed based on Equation (6).

## 6 CONCLUSION

We have developed a new method that enables variable anisotropic FOV for 3DPR with spiral phyllotaxis. Based on the user defined anisotropic FOV, the corresponding radial trajectories can be generated on the fly because the computational demand for the proposed method is very small. The proposed method can reduce imaging times and/or minimize aliasing artifacts depending on the anisotropy of the object dimensions within the excited imaging volume.

## CONFLICT OF INTEREST

All the authors are employed by Philips Healthcare.

## Supporting information


**FIGURE S1** The efficiency of VASP changes with the FOV anisotropy ratio. The plot shows the percentage of radial projections required in VASP when compared to the conventional SP as a function of the ratio between the desired *FOV* in *z* and *FOV* in *xy* (*x* = *y*) directions respectively. Note: The number of radial projections required for conventional SP was computed using VASP with a fixed FOV ratio of 68:68:100 (*x*:*y*:*z*)
