# Supplementary material for: Variable anisotropic FOV for 3D radial imaging with spiral phyllotaxis (VASP)
Source: Magn Reson Med. 2020 Aug 27;85(1):68–77. doi: 10.1002/mrm.28449 (PMC7692914; doi:10.1002/mrm.28449)
Supplement: Supplementary file 1 — FIGURE S1 The efficiency of VASP changes with the FOV anisotropy ratio. The plot shows the percentage of radial projections required in VASP when compared to the conventional SP as a function of the ratio between the desired FOV in z and FOV in xy (x = y) directions respectively. Note: The number of radial projections required for conventional SP was computed using VASP with a fixed FOV ratio of 68:68:100 (x:y:z) [file MRM-85-68-s001.docx]

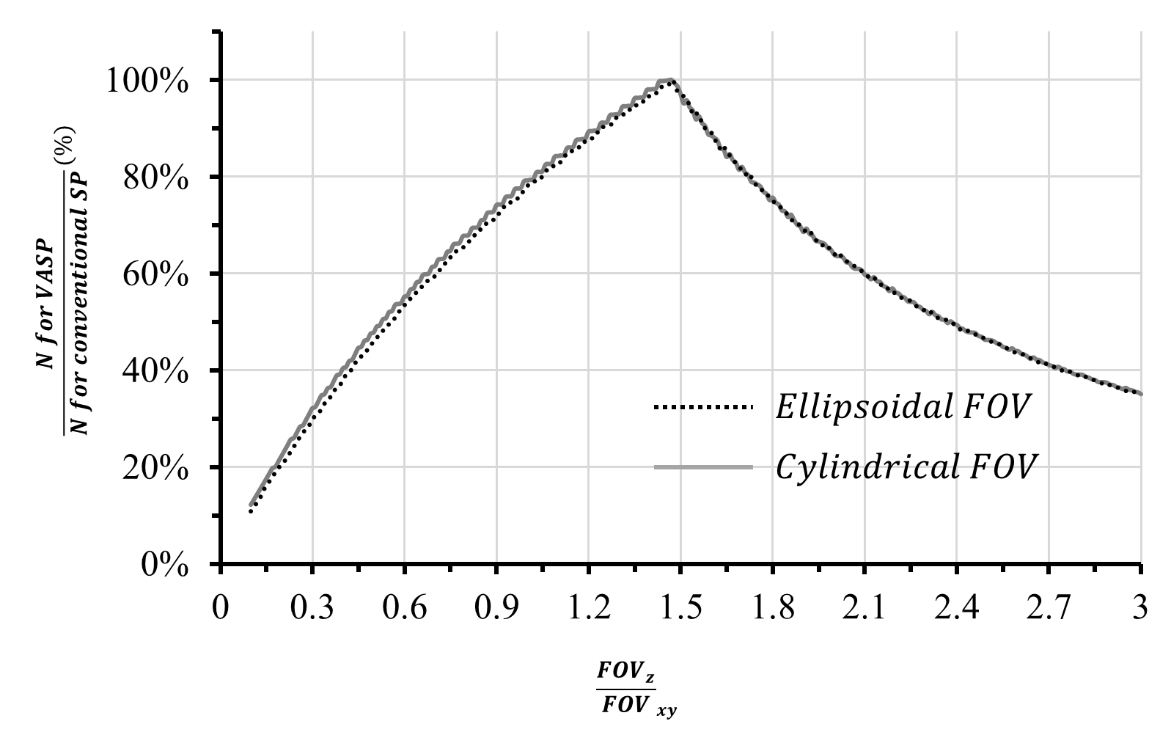


Supporting information figure S1. The benefit of VASP changes with the FOV asymmetry. The plot shows the percentage of radial projections required in VASP when compared to the conventional SP as a function of the ratio between the desired$FOV$in $z$ and $FOV$in $xy$ ($x=y$) directions respectively. Note: The number of radial projections required for conventional SP was computed using VASP with a fixed FOV ratio of 68:68:100 $\left( x :y :z \right)$.
